# Progression Patterns in Non-Contrast-Enhancing Gliomas Support Brain Tumor Responsiveness to Surgical Lesions

**DOI:** 10.3389/pore.2022.1610268

**Published:** 2022-05-30

**Authors:** Steffen Brenner, Sebastian Hartzendorf, Philip Vogt, Elena Maier, Nima Etminan, Erik Jung, Wolfgang Wick, Felix Sahm, Frank Winkler, Miriam Ratliff

**Affiliations:** ^1^ Department of Neurosurgery, University Hospital Mannheim, University of Heidelberg, Mannheim, Germany; ^2^ Neurology Clinic and National Center for Tumor Diseases, University Hospital Heidelberg, Heidelberg, Germany; ^3^ Clinical Cooperation Unit Neurooncology, German Cancer Consortium (DKTK), German Cancer Research Center (DKFZ), Heidelberg, Germany; ^4^ Department of Neuropathology, University Hospital Heidelberg, University of Heidelberg, Heidelberg, Germany; ^5^ Clinical Cooperation Unit Neuropathology, German Cancer Consortium (DKTK), German Cancer Research Center (DKFZ), Heidelberg, Germany

**Keywords:** tumor progression, astrocytoma, surgical lesioning, tumor cell network, tumor microtubes

## Abstract

**Purpose:** The overall benefit of surgical treatments for patients with glioma is undisputed. We have shown preclinically that brain tumor cells form a network that is capable of detecting damage to the tumor, and repair itself. The aim of this study was to determine whether a similar mechanism might contribute to local recurrence in the clinical setting.

**Methods:** We evaluated tumor progression patterns of 24 initially non-contrast-enhancing gliomas that were partially resected or biopsied. We measured the distance between the new contrast enhancement developing over time, and prior surgical lesioning, and evaluated tumor network changes in response to sequential resections by quantifying tumor cells and tumor networks with specific stainings against IDH1-R132H.

**Results:** We found that new contrast enhancement appeared within the residual, non-enhancing tumor mass in 21/24 patients (87.5%). The location of new contrast enhancement within the residual tumor region was non-random; it occurred adjacent to the wall of the resection cavity in 12/21 patients (57.1%). Interestingly, the density of the glioma cell network increased in all patient tumors between initial resection or biopsy and recurrence. In line with the histological and radiological malignization, Ki67 expression increased from initial to final resections in 14/17 cases.

**Conclusion:** The non-random distribution of glioma malignization in patients and unidirectional increase of anatomical tumor networks after surgical procedures provides evidence that surgical lesions, in the presence of residual tumor cells, can stimulate local tumor progression and tumor cell network formation. This argues for the development of intraoperative treatments increasing the benefits from surgical resection by specifically disrupting the mechanisms of local recurrence, particularly tumor cell network functionality.

## Introduction

Gliomas are the most common primary brain tumors. There is a strong body of evidence on the relation of surgical resection as first-line as well as recurrence treatment and improved overall survival and progression free survival. Further, several studies underlined that a gross total resection is superior over subtotal resection or biopsy in newly diagnosed lower-grade glioma and glioblastoma (1–5). A marked survival advantage in favor of early resection in cases with suspected lower-grade glioma was also demonstrated in a paper comparing opposite surgical management strategies at two Norwegian University Hospitals (6). Data from various studies emphasize complete resection—whenever safely possible—as one of the strongest, independent predictors of not only overall survival but also malignant transformation rate and the degree of seizure control in patients with lower-grade glioma (6–9). Even subtotal resections might still be of clinical value (10).

Despite the invasive nature of glioblastoma, recurrence of this astrocytic primary brain tumor occurs at the resection margin in 90% of patients (11). This observation has been attributed to residual tumor cells that surround the resection margin in a tapering gradient. The presumption is that residual tumor cells are generally equipotent in their tumorigenicity and that higher density at the resection margin increases the probability that new growth will occur there. This notion has remained largely unchallenged for decades. However, recent reports have generated support for the contribution of a wound healing-like response to brain tumor recurrence (12, 13).

We recently reported that astrocytic tumor cells interconnect through long membrane protrusions called tumor microtubes to form a functional network. We have shown that this network mediates brain invasion and tumor progression and contributes to resilience to chemo- and radiotherapy (13–16). We found that damage to tumors inflicted through surgical lesioning cause “malignant repair”—replacement of resected tumor cell components that eventually exceed the density of the initial tumor network (13). It is difficult to comprehend that glioma surgery, a medical procedure that has an important and beneficial role in the treatment of glioma (17, 18), could contribute not just to the relapse but also to the progression towards a more malignant state of the disease it was intended to treat (19).

**FIGURE 1:**
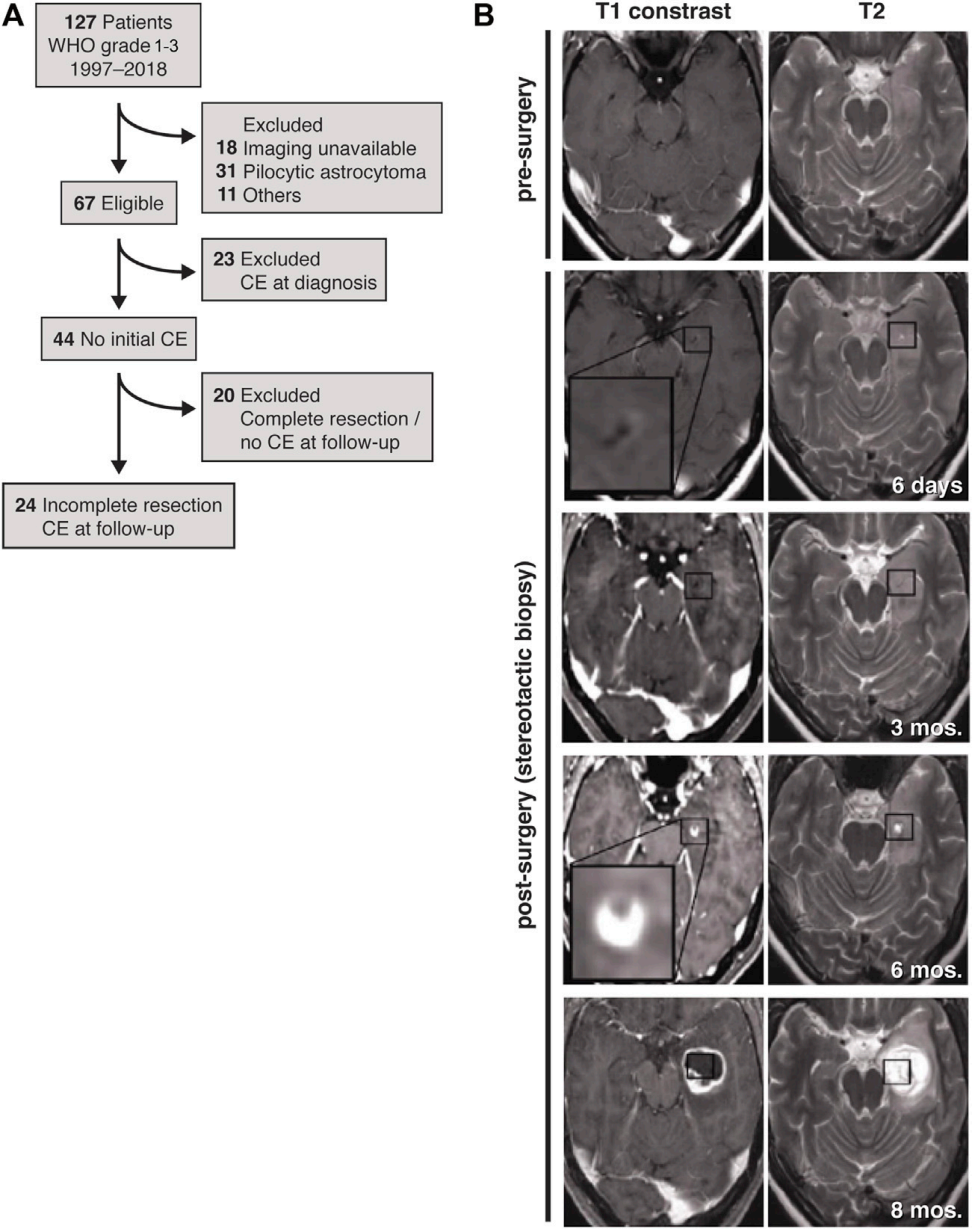
Study design and representative MR images. **(A)** Patient flow diagram for inclusion and exclusion of patients with initially non-contrast enhancing astrocytoma that developed contrast enhancement during the course of disease. **(B)** Before surgery (first row), the initial tumor is non-contrast-enhancing and is only seen in the T2 weighted MRI (first row, right image). After stereotactic biopsy the lesion is discernible within residual tumor (second row). Post-surgery, follow-up T1-weighted MRIs reveal initial contrast enhancement at the lesion border after 6 months (fourth row) with obvious tumor progress 8 months after stereotactic biopsy (fifth row). Axial planes are shown.

However, in consideration of all these data, the effect of the surgical lesion on the stimulation of a tumor self-repair response resulting in tumor malignization itself may require a deeper understanding. We retrospectively studied glioma progression to compare the contributions of residual tumor cells and surgical lesioning to secondary malignization and tumor progression. Thus, we strategically restricted our patient cohort to cases of partial resection and stereotactic biopsy (Figure 1A). These criteria enabled us to analyze tumor progression with respect to the resection cavity and regions of residual, non-resected tumor cells.

## Materials and Methods

### Patients

We identified patients diagnosed between January 1997 and May 2018 with non-contrast-enhancing gliomas at the University Medical Center Mannheim. From this patient cohort, we identified 24 patients with partial resections or lesions from stereotactic biopsies that were non-contrast-enhancing, T2-FLAIR (T2-weighted fluid-attenuated inversion recovery) hyperintensive and that progressed to a secondary contrast-enhancing tumor (Figures 1A,B; Supplementary Figure S1). We did not find patients with oligodendrogliomas and decided to exclude them from this study, since tumor microtubes are rarely found in this entity.

Because of the long time span of our study, molecular sample analysis was only partly available for the patients. All tumor entities included in the study are known to extend long membrane tubes and form a functional communicating multicellular network. The diagnosis of astrocytoma was confirmed in all cases, using histological criteria, and immunohistochemical staining for IDH1-R132H, with IDH mutation found in 11/24 cases. ATRX nuclear staining was performed in all patients, too, and an ATRX loss was confirmed in 21/24 patients. Loss of ATRX expression in the presence of an IDH mutation generally excludes 1p/19q loss of heterozygosity and therefore a diagnosis of oligodendroglioma (which are generally IDH mutated) (20, 21).

The study protocol conformed to the ethical guidelines of the Declaration of Helsinki and was approved by the institutional ethical review board of Heidelberg University (2018-843R-MA and 2018-614N-MA).

### Extent of Resection and Malignization

All patients included in the present analysis received early postoperative MRI within 72 h after surgery to determine the extent of resection. Remaining postoperative T2-FLAIR hyperintensity was considered residual tumor. Evaluation of the extent of resection was performed by comparing segmented pre- and postoperative T2-FLAIR hyperintensity images side by side (Brainlab Elements, Brainlab AG, Munich, Germany). We defined partial resection with a cut-off level of ≤95% based on the T2-FLAIR images.

Malignization was defined as new contrast enhancement during follow-up MRIs. We stratified new contrast enhancement into defined recurrence patterns based on their absolute distances from the resection cavity: regional (in the wall of the resection cavity), marginal (≤20 mm of the resection cavity) and distant (>20 mm from the resection cavity); The absolute distance was measured from the border of the contrast enhancement to the resection cavity in either the axial, coronal or sagittal plane, in whichever plane the distance was shortest. A “multiple” category was also generated to designate the incidence of more than one focus of contrast enhancements—in such cases, the contrast enhancement nearest to the resection cavity was measured (Table 1). To account for the respective variations in residual tumor volume among the 21 patients that developed a new contrast enhancement within the residual tumor, we also described the distance between contrast enhancement and the resection cavity in relationship to the size of the residual tumor (Table 1). Therefore, contrast enhancement distances are described relatively, as a ratio of their distance from the resection cavity to the length of the residual tumor within the same MRI plane.

**TABLE 1:** Patient and tumor characteristics.

Patient	Age	Sex	Tumor location	Postoperative tumor		Adjuvant therapy	Distance between CE and RC		Recurrence pattern^c^	Time to CE (months)^d^
				Volume (ml)	EOR (%)		Absolute (mm)^a^	Relative^b^		
MA01	32	M	left temporal	26.3	biopsy	R	0.0	0.00	Regional	66
MA02	10	F	right temporal	79.6	20	R + TMZ	3.5	0.21	Multiple	11
MA03	4	M	pontine	42.7	biopsy	R + TMZ	0.0	0.00	Regional	1
MA04	30	F	left temporal	70.4	40	R	14.0	0.39	Marginal	70
MA05	39	M	left frontal	164.6	20	R + PCV	6.5	0.41	Marginal	48
MA06	29	M	right frontal	22.3	27	R + TMZ	0.0	0.00	Regional	20
MA07	59	M	right temporal	43.6	57	none	7.9	0.42	Marginal	49
MA08	33	M	left temporal	87.2	46	none	0.0	0.00	Regional	31
MA09	52	F	left parietal	6.1	biopsy	none	0.0	0.00	Regional	43
MA10	44	F	left parietal	27.9	10	none	0.0	0.00	Regional	137
MA11	62	F	right parietal	43.5	biopsy	R + TMZ	31.0	0.65	Distant	35
MA12	28	F	right temporal	32.8	85	R + TMZ	8.9	0.30	Marginal	10
MA13	36	F	left frontal	12.8	95	none	0.0	0.00	Regional	55
MA14	74	M	right frontal	49.4	13	R	1.5	0.28	Marginal	10
MA15	53	M	left frontal	52.2	biopsy	none	1.0	0.02	Multiple	13
MA16	47	M	left parietal	12.4	26	none	0.0	0.00	Regional	57
MA17	49	M	right temporal	25.2	18	none	0.0	0.00	Regional	14
MA18	68	F	bifrontal	19.4	biopsy	none	13.6	0.38	Marginal	4
MA19	76	F	right temporal	66.8	16	R + TMZ	0.0	0.00	Multiple	10
MA20	37	M	left temporal	52.2	65	none	0.0	0.00	Regional	57
MA21	32	F	right frontal	49.0	43	R + TMZ	0.0	0.00	Regional	26
MA22	20	F	right frontal	14.4	93	R + TMZ	26.1	n/a	Distant	30
MA23	65	M	right parietal	26.1	biopsy	R + TMZ	26.9	n/a	Distant	12
MA24	40	M	right temporal	54.1	31	R + TMZ	10.9	n/a	Marginal	19

CE, contrast enhancement; EOR, extent of resection; n/a, not applicable; PCV, procarbazine + lomustine (CCNU) + vincristine; R, radiotherapy; RC, resection cavity; RT, residual tumor; TMZ, temozolomide.

a The absolute distance was measured from the border of the CE to the resection cavity.

b To compensate for variations in residual tumor volume we also determined the distance between CE and the RC relatively to the size of the RT. Relative distance is the ratio of absolute distance to the respective length of the RT. For example, CE developing at the wall of the RC has a relative distance of 0.0. In contrast, a new CE located at the maximal distance from the RC, but still within the RT, has a relative distance of 1.0.

c Patterns of recurrence were categorized as regional (in the wall of the RC), marginal (≤20 mm of the RC), distant (>20 mm from the RC), or multiple (multiple foci of CE). Categories were adapted from Konishi et al. (25).

d Time to CE is the time period from the resection to the MRI scan that first showed CE.

Histological reassessment was performed if and when patients underwent another surgery after development of a contrast enhancement. The primary endpoint within this study was onset of a new contrast enhancement; the secondary endpoint was overall survival.

### Determination of Histological Malignization and Glioma Network Increase

The Department of Neuropathology at Heidelberg University provided immunohistochemical staining of paraffin-embedded tissue sections, using DAB to reveal the respective primary antibodies to IDH1-R132H (DIA-H09, Dianova, RRID: AB_2335716), ATRX (HPA 001906, Sigma-Aldrich, RRID: AB_1078249) and Ki67 (ab15580, Abcam, RRID: AB_443209) and using hematoxylin as counterstain. To analyze stained sections, these were first digitized and converted into RGB TIFF files with an Axio Scan.ZI slide scanner controlled by ZEN software (Zeiss).

Quantification of IDH1-R132H-positive staining was performed computationally with a Fiji image-analysis software (22) macro using three representative 250 µm-square regions per tumor sample. Specifically, IDH1-R132H staining was separated from hematoxylin staining with the color deconvolution plugin using the built-in “H DAB” vector settings. This separated DAB-only layer represented overall IDH1-R132H staining. Then an overlay of quantifiable false-colored pixels (binary—stained vs. unstained pixels) was created by applying the default iterative threshold algorithm using a minimum-threshold value set to zero and a maximum-threshold value set to an 8-bit number (0–255) in which visually stained tissue was optimally segmented from unstained tissue; the sum of the pixels from this overlay quantified total staining. A second overlay from the DAB-only layer was created in which cell body staining was segmented from cytoplasmic tumor microtube density, again using the default threshold algorithm and zero minimum threshold, but adjusting the maximum threshold value to an 8-bit number in which only cell bodies were visually segmented; the sum of the pixels from this overlay quantified cell body staining. The difference between the sums of the two overlays gave the value for net tumor microtube network density. To compensate for variations in the number of tumor cells per region, cell bodies were counted and then the average net tumor microtube network density per tumor cell was calculated by dividing net tumor microtube network density by the total number of cell bodies. The results of the three representative regions were averaged to compensate for sample variation.

This entire process of quantifying net tumor microtube density was performed and evaluated independently by two individuals (SB, MR) to offset inter-rater variability. See Supplementary Figure S2 for an illustration of the quantification process.

Ki67 is a proliferation marker that we used to compare the malignization of tumors between sequential resections.

### Statistical Analysis

Analyses were conducted using SigmaPlot 14.0 (SYSTAT). Two-by-two tables were constructed and analyzed using Fisher’s exact test to test if there is statistical significance that patients without adjuvant therapy mostly showed new contrast uptake regional or marginal. Using the two-tailed Mann-Whitney U test, *p*-values were determined from changes in Ki67 relative count and IDH1-R132H net tumor microtube network density during the progression of the disease. Statistical significance was set to *p* < 0.05.

## Results

### Characteristics of Patient Cohort and Tumors

As a result of our inclusion and exclusion criteria, out of 127 patients with WHO grade 1–3 gliomas, ultimately 24 patients were included (Figure 1A), of which 17 patients underwent partial resections and seven patients received stereotactic biopsies only of a non-contrast-enhancing astrocytoma (Table 1). Twenty-one patients were previously diagnosed as lower-grade astrocytoma (WHO grade 2–3). One additional patient was initially diagnosed under guidance of 2007 WHO classification as “oligoastrocytoma”, but has since been revised to astrocytoma (MA17; Figure 4A). Two patients initially did not reveal a clear pathological diagnosis after stereotactic biopsy of a non-contrast-enhancing T2-FLAIR hyperintensive lesion.

Patients that underwent open surgery with planned partial tumor resection received a median 42 ± 27 (s.d.) percent tumor resection. The median follow-up period for the patients was 68.5 ± 56.9 (s.d.) months (range: 12–193 months) (Table 1).

A novel contrast enhancement was seen during follow-up at a median contrast enhancement onset of 35 ± 30 (s.d.) months (range: 1–137 months) (Table 1). Patients developing an early, new contrast enhancement did not undergo immediate re-resection until a follow-up MRI confirmed suspected contrast enhancement progression. Seventeen patients underwent a re-resection 29 ± 24 (s.d.) months after their first surgery (range: 1–81 months). Pathologists concluded that samples from all seventeen re-resections were derived from solid tumors that had increased at least one WHO classification grade (Figure 4A); pseudoprogression was not diagnosed in any of the cases.

### Patterns of Malignization Suggest Procedure-Related Factors

We reasoned that if the remaining tumor load solely accounts for recurrence and malignization, the distribution of new contrast enhancement within the residual tumor regions of patients would be stochastic (Figure 3A). Alternatively, if surgical lesions instigate secondary malignization, contrast enhancement would develop predominantly within the direct vicinity of the resection cavity (Figure 3B).

**FIGURE 2:**
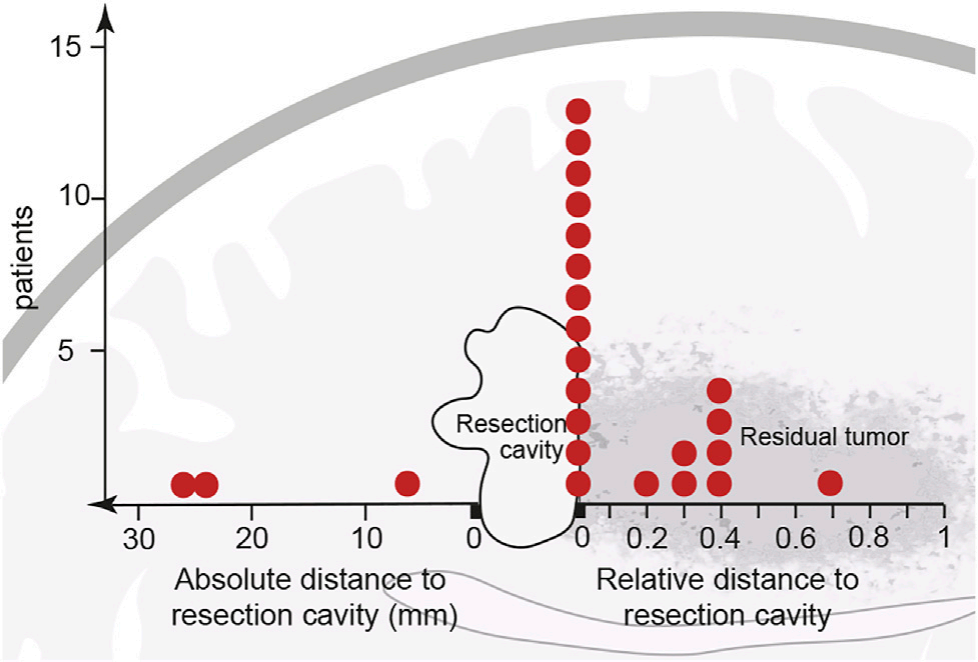
Initial location of newly developed contrast enhancement in relation to resection cavity and main tumor area. After partial resection or biopsy of a previously non-contrast-enhancing tumor, 21 of the 24 cohort patients developed contrast enhancement within the residual tumor shown adjacent to the resection cavity. Thirteen patients developed contrast enhancement in the direct vicinity of the resection border. Eight other patients developed contrast enhancement at a distance from, but still within, the residual tumor; however, because every residual tumor size is unique, contrast enhancement distances are described relatively, as a ratio of their distance from the resection cavity to the length of the residual tumor. Of the 24 patients only three developed contrast enhancement outside the residual tumor (data points to the left of the resection cavity). Each red dot represents one patient.

**FIGURE 3:**
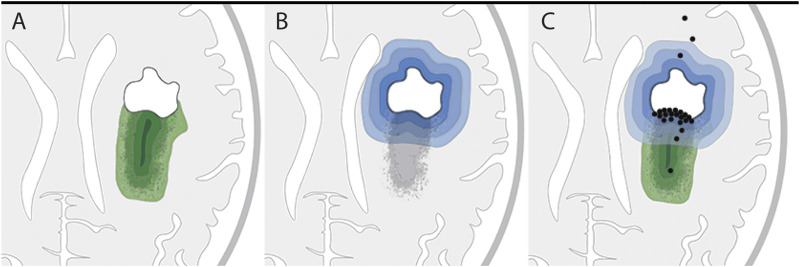
Hypothetical versus actual contrast enhancement after surgical lesioning. **(A,B)** Two hypothetical models of tumor progression: **(A)** Residual tumor model: residual tumor seeds tumor recurrence, with higher probability of recurrence within tumor regions of higher cell density (increasingly darker green layers). **(B)** Surgical lesioning model: tumor recurrence is initiated in response to wounding at the site of surgical lesioning; probability of recurrence gradually decreases as distance from the resection cavity increases (increasingly lighter blue layers). **(C)** Illustration of actual locations of initial contrast enhancement (black dots) from the 24 patients in this study in relation to the residual tumor. Twenty-one patients developed initial contrast enhancement within the residual tumor tissue; of these 21 patients, 12 had contrast enhancement in the direct vicinity of the resection border. The locations of initial contrast enhancement of three patients were outside the residual tumor and distant to the border of the resection cavity.

The follow-up MRIs indicated that, with only a few exceptions, new contrast enhancement emerged from within residual tumor regions (21/24 patients, 87.5%) (Figures 1B, 2; Supplementary Figure S1). The distribution of new contrast enhancement within residual tumor regions was not stochastic, with the majority of new contrast enhancement clearly developing at the resection cavity (Figure 2; right x-axis, Figure 3C). Our measurements revealed that new contrast enhancement occurred directly adjacent to the resection cavity in 12 patients (57.1%) and within 1 cm of the resection cavity in six patients (28.6%) (Figures 2, 3C; Table 1).

In sum, 11 new contrast enhancements were regional, seven marginal, three distant and three were multiple. Among the multiple contrast enhancement recurrence pattern cases, patient MA02 developed two contrast enhancement foci within the residual tumor and both fell into the marginal class. Patient MA15 developed four contrast enhancement foci: three were marginal and within the RT; one was distant. Patient MA19 developed two contrast enhancement foci: one CE was regional and within the residual tumor; one was distant, likely due to subependymal spread. (Supplementary Figure S1; in cases of multiple contrast enhancements, only MRI data of the nearest contrast enhancement are shown).

Considering the size of the remaining tumor at index surgery the measurement of relative distances to the resection cavity (Table 1) demonstrated, that 13 patients developed contrast enhancement at a relative distance of close to zero, seven patients developed contrast enhancement within half the distance of the residual tumor, while only one developed contrast enhancement within more than half the distance of the residual tumor (Figure 2; right x-axis).

In only three cases from our patient cohort (12.5%) contrast enhancement developed elsewhere in the brain, outside of the previously T2-FLAIR hyperintensive region. The contrast enhancement in these cases was located 10–30 mm from the resection cavity (Figure 2; left x-axis, Table 1).

In the context of tumor recurrence, within the group of patients with distant and multiple contrast enhancements, four out of those six received additional adjuvant therapy, which has been shown as a trigger of tumor self-repair in preclinical studies (13, 14). Patients without adjuvant therapy mostly developed new contrast uptake regionally or marginally (9 of 10 cases); however, this observation did not reach statistical significance (*p* = 0.36).

### Surgical Lesioning Is Accompanied by Increased Tumor Microtube Density

**FIGURE 4:**
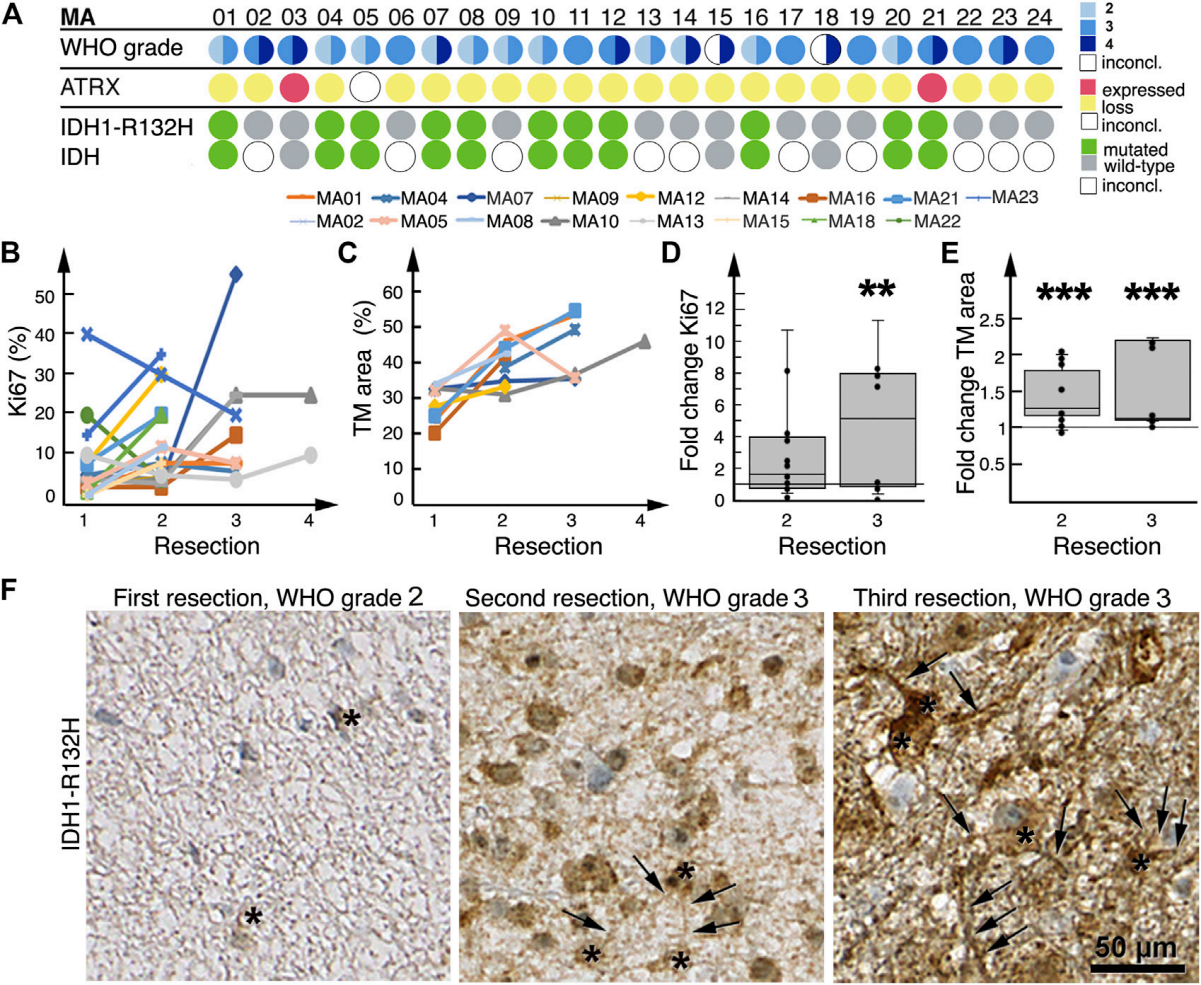
Histological malignization and tumor microtube network increase at recurrence. **(A)** Summary of patient tumor classification. Below the row of patient numbers, the corresponding results from WHO grading as diagnosed by neuropathologists at the time of resection, ATRX and IDH1-R132H staining of patient tumors are shown. Tissue sections were only stained for IDH1-R132H, other rarer IDH mutations might have well been overlooked as molecular diagnosis was only available for MA03, MA15 and MA18. WHO grading circles are divided into two halves; the left half represents initial tumor diagnosis, the right half represents tumor diagnosis after the last resection. For patients who had only one resection or biopsy, the circles are not divided. For two patients the initial diagnosis was inconclusive. **(B)** Tumor expression of Ki67 at sequential resections. **(C)** Tumor microtube area in tumors at sequential resections. **(D)** Fold change in Ki67 expression at second and third resections relative to first resection, respectively. **(E)** Fold change in total tumor microtube area at second and third resections relative to first resection, respectively. Boxplots in both **(D,E)** show median, first quartile and third quartile values. **p = 0.008; ***p ≤ 0.001. **(F)** IDH1-R132H-stained tumors from patient MA01 after first, second and third resections; total tumor microtube area (arrows) increases over time. Asterisks identify IDH1-R132H-positive tumor cells.

We detected an increase in Ki67 expression from initial to final resections in 14/17 patient tumors (Figure 4B), which was in line with their histological and radiological malignization (Figure 4A). Between first and second resections, fold change in Ki67 positivity was a median of 1.6 (range: 0.2–3.8); between first and third resections, fold change was a median of 5.0 (range: 0.4–11.0, *p* = 0.008) (Figure 4D).

Eleven patient tumors harbored a IDH1-R132H mutation (Figure 4A), which allowed for a tumor cell-specific staining of tumor microtubes and their cell bodies (14, 16, 23) (Figure 4F). Nine of these 11 patients had more than one resection; we found that in all nine patient tumors, total tumor microtube area increased between sequential resections (Figures 4C, F). In relation to first resections, total tumor microtube area increased 1.36-fold in second resections (0.95–1.96) (*p* = 0.001) and 1.11-fold in third resections (1.08–2.16) (*p* ≤ 0.001), respectively (Figure 4E).

## Discussion

Despite the propensity for glioma cells to diffusely colonize the entire brain (24), astrocytic glioma typically recur locally at the very place of the resection cavity. In an attempt to define a potential contribution of surgical lesioning for glioma recurrence in patients, potentially by a tumor self-repair mechanism recently discovered by our group (13, 14), we retrospectively analyzed non-contrast-enhancing astrocytoma that showed secondary contrast enhancement after partial resection or biopsy. Our study aimed to describe recurrence patterns compatible with the concept of a malignant repair process in patients.

Traditionally, remaining tumor cells have been held accountable for the predominantly local recurrence pattern after surgery in glioblastomas (11, 25, 26). This assumption is in line with the correlation of the extent of tumor resection and the progression-free as well as overall survival (1, 27). Our patient cohort was assembled based on strategic criteria that should enable discrimination between local effects induced by surgical lesioning and/or post-surgical inflammation as well as the role of the remaining tumor cell load. Should the tumor cell load be solely responsible for tumor progression, secondary malignization should have been observed stochastically anywhere within the remaining T2-FLAIR hyperintensive tumor. We found that 21/24 patients developed contrast enhancement within residual tumors, however the majority of contrast enhancement was in direct or close proximity to the resection cavity suggesting that the presence of residual tumor cells is necessary but insufficient to explain recurrence at resection margins.

We thus propose that biological mechanisms of glioma recurrence include surgical lesioning. The data within in this study suggest that tumor network density increases in recurrent astrocytomas between serial resections, providing translational supportive evidence that tumor microtubes contribute to the lesioning response and potentially in the end to the malignization process.

While various additional mechanisms of glioma resistance to chemo- and radiotherapy have been observed and studied for years (28–30), the impact of surgical lesioning on the microenvironment at the resection border and its effect on local tumor recurrence has received little attention in the past. Preclinical research has implicated both normal and tumor cells in a repair mechanism that triggers tumor progression. The response to a variety of pathological stimuli in the brain involves reactive astrocytes, the main cellular component of glial scars. Okolie et al. (12) have shown that surgical lesioning is accompanied by reactive astrocytosis within the local peritumoral microenvironment. These astrocytes, when cocultured with tumor cells, promote tumor cell proliferation and migration (12). In gliomas studied orthotopically in mice we recently identified that surgical scarring triggers an excessive tumor repair mechanism that increases the complexity of cell-cell interconnectivity and recruits tumor cells to the resection border, thereby promoting local tumor progression (12, 13). Tumor cells extend tumor microtubes toward the resection cavity to repair the lesion and reconstruct the integrity of the tumor network, finally resulting in an overshooting “repair response” with more tumor cells in the area of the resection margin than before (13).

Our data within this study sustain a corresponding lesioning-directed build-up of the tumor network apparatus supporting the hypothesis that local glioma recurrence can result from a response to resection-induced tissue damage that is exploited by astrocytic tumors to repair and further fortify itself by producing more tumor microtubes. This finding suggests that the preclinical findings of tumor cell network self-repair might indeed be relevant for patients.

The small number of patients is a limitation of our study; another restraint comes with the retrospective nature and the lengthy clinical course that limits detailed information on the exact spatial location of the resected recurrent disease compared to the tissue analyzed by histology at the index surgery. Molecular sample analysis was only partly available for the tissue sections. We were able to add immunohistochemical staining for IDH1-R132H and ATRX nuclear staining but did not exclude other IDH mutations. But importantly, despite the molecular heterogeneity of the tumor samples within our patient cohort, functional tumor microtubes networks are consistently present in all human brain tumor material of incurable glioma types (14, 16, 23, 31), even including H3K27 mutated diffuse midline childhood glioma (31). Furthermore, it cannot be excluded that the *a priori* selection of the biopsy or resection site has a positive predictive effect on the site of recurrence near this site. Despite these limitations this is to our knowledge the first study that analyzed the patterns of contrast enhancement in partially resected previously non-contrast enhancing astrocytic gliomas.

Our study does not challenge the role and relevance of surgical reduction of gliomas as one mainstay of therapy, with overall beneficial effects for patients, - but rather highlights the need for novel and local treatment strategies that prevent formation of a recurrence-prone microenvironment at the resection cavity. It is plausible to assume that specific inhibition of tumor microtube formation by postsurgical targeting of its key drivers like GAP-43 (13) could inhibit local recurrence after surgery. This can also include inhibitors of main activators of tumor cell networks, such as the neuron-glioma synapses (16). It has been demonstrated that the interference with glutamatergic AMPA-receptors by perampanel comprises the functionality of the neuron-glioma synapses that normally stimulates the maintenance of glioma cell networks (16, 31). Thus, the data presented here further support the development of additional local or systemic perioperative therapies, preferentially aimed at GAP-43, the neuron-glioma synapses or other molecular drivers of the malignant tumor cell networks (13–16). A clinical trial with perioperative administration of perampanel and careful molecular and longitudinal radiological studies is planned to build on this observational data and the preclinical proof-of-principle.

## Data Availability

The original contributions presented in the study are included in the article/Supplementary Material, further inquiries can be directed to the corresponding author.
