# Supplementary material for: Progression Patterns in Non-Contrast-Enhancing Gliomas Support Brain Tumor Responsiveness to Surgical Lesions
Source: Pathol Oncol Res. 2022 May 30;28:1610268. doi: 10.3389/pore.2022.1610268 (PMC9189286; doi:10.3389/pore.2022.1610268)
Supplement: Supplementary file 1 [file DataSheet2.PDF]

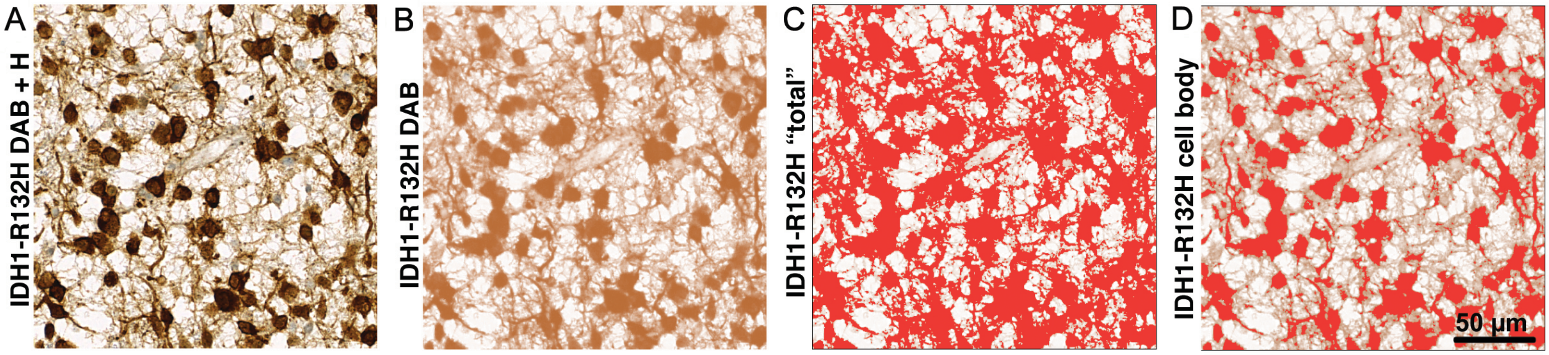

Supplementary Figure 2
Illustration of the process of quantifying tumor microtube (TM) area from a digitized image of a paraffin section that was immunostained with IDH1-R132H and counterstained with hematoxylin. This representative section was derived from patient MA02; the tumor was histologically diagnosed as a WHO grade 3 astrocytoma. (A) A 250 µm-square region of a scanned stained section. (B) DAB-only layer after color deconvolution using the "H DAB" vector setting of the Fiji color deconvolution plugin; the DAB-only layer represents overall IDH1-R132H staining. (C) An overlay of quantifiable red pixels, created by applying a threshold to the DAB-only layer in which stained regions were optimally segmented from unstained regions; the sum of red pixels quantifies total IDH1-R132H staining. (D) A second overlay of quantifiable red pixels, which correspond to cell body staining, was created by applying a threshold in which cell bodies were visually segmented from TM network area. The net TM network area was then calculated by subtracting the quantification of cell body staining (D) from total staining (C). To compensate for variation in the number of tumor cells per region, we also counted tumor cell bodies and then calculated the average TM network area per tumor cell.
