# Supplementary material for: Progression Patterns in Non-Contrast-Enhancing Gliomas Support Brain Tumor Responsiveness to Surgical Lesions
Source: Pathol Oncol Res. 2022 May 30;28:1610268. doi: 10.3389/pore.2022.1610268 (PMC9189286; doi:10.3389/pore.2022.1610268)
Supplement: Supplementary file 2 [file DataSheet1.PDF]

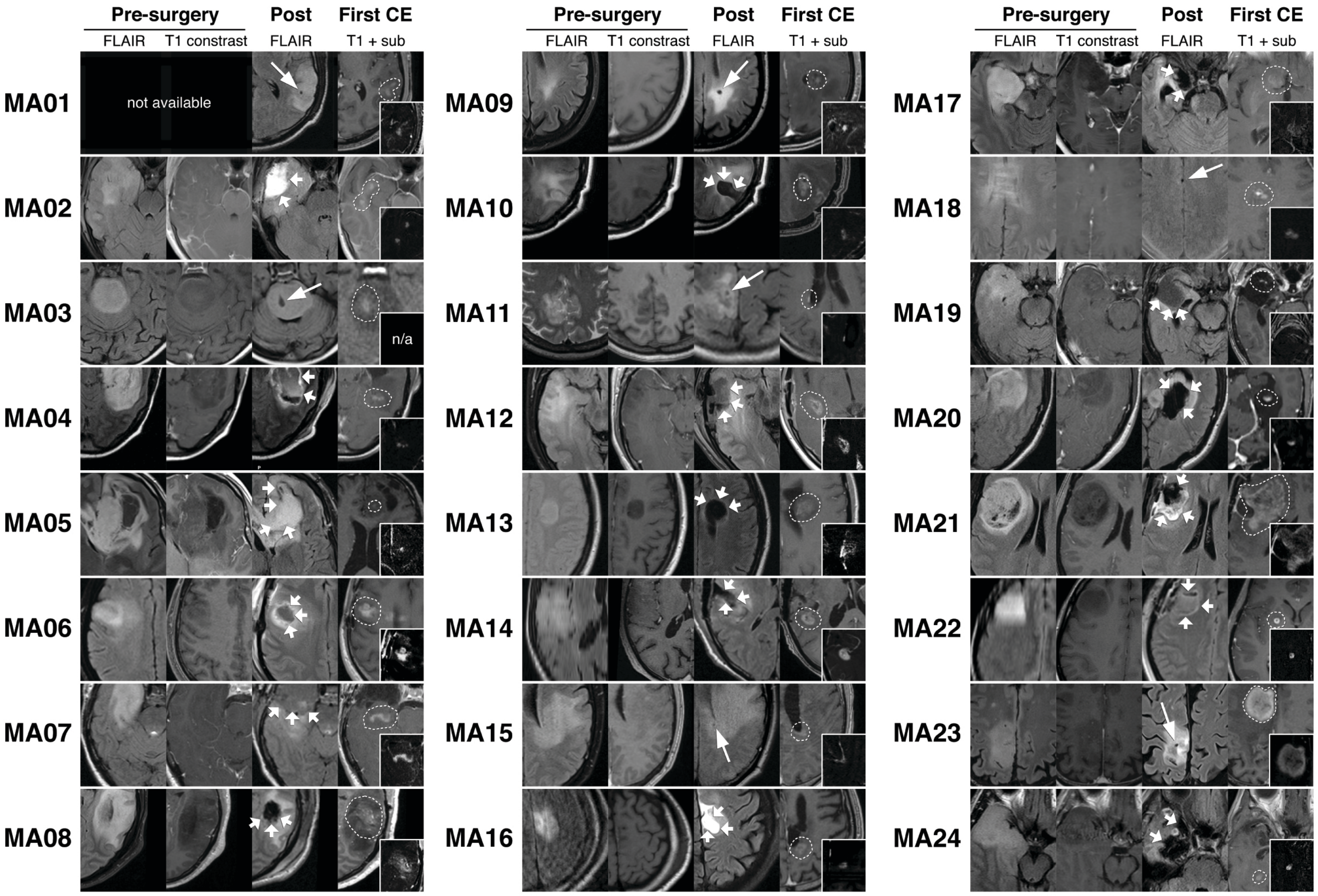

Supplementary Figure 1
MRIs from patient cohort before and after surgical resection. Before surgery, the initial tumor is only seen in the T2-fluid-attenuated inversion recovery (T2-FLAIR) image (first column from left). No contrast enhancement (CE) was observed just prior to surgery (second column). After stereotactic biopsies or partial resections, resection cavities are discernible (third column). Post-surgery, follow-up T1-weighted MRIs reveal initial CE (fourth column). Subtraction images, unenhanced T1-weighted MRIs from contrast-enhanced MRIs, emphasize tumor CE (fifth column).
